# Optical characteristics of laser medical instrument with side-firing fiber under complete bevel angle range

**DOI:** 10.1016/j.isci.2024.110769

**Published:** 2024-08-20

**Authors:** Diqing Ying

**Affiliations:** 1 College of Optical Science and Engineering, Zhejiang University, Hangzhou 310027, China

**Keywords:** Health sciences, Natural sciences, Physics

## Abstract

The side-firing instrument is studied under the complete bevel angle range. The fiber core and cladding are 0.6 mm and 0.66 mm, respectively, the fiber core refractive index is 1.457, and the fiber cladding refractive index is from 1.409 to 1.452 corresponding to the numerical aperture from 0.37 to 0.12. The bevel angle range is subdivided by ten crucial angles, whose relationship changes as the fiber cladding refractive index reaches 1.418. The beam’s divergence angle and coverage increase as the bevel angle deviates from being equal and close to π/4 rad, respectively. When all rays achieve total internal reflection, with numerical aperture being 0.37, the divergence angle and coverage would increase by 28.29% and 44.74%, respectively. The required emission opening size has a minimum under the bevel angle being close to π/4 rad, whose expression is obtained. It increases sharply as the bevel angle reaches a certain value.

## Highlights

• Complete bevel angle range is subdivided by ten crucial angles
• Divergence and coverage increase sharply as bevel angle reaches a certain value
• Expression of required emission opening size is obtained
• Emission opening size increases sharply as bevel angle reaches a certain value

## Graphical abstract

**Figure:**
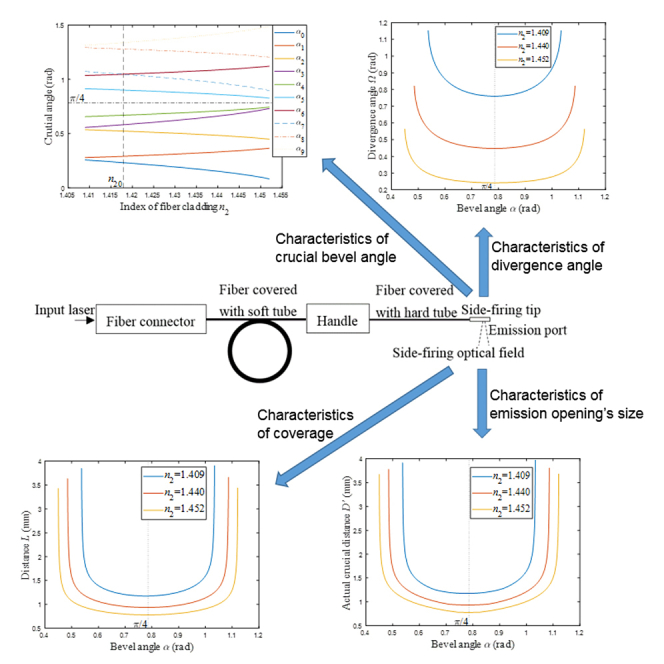

## Introduction

The endoscopic laser treatment may offer a valuable alternative to the traditional operation procedure in such as the removal of glottis lesions, since it has several advantages, such as the rapid sealing of vascular tissues.^1^^,^^2^^,^^3^ The instrument of this treatment generally involves the flexible optical fiber to deliver the laser aimed directly at the abnormal tissue, and it generally includes two types.^1^^,^^4^ One type of this instrument utilizes the traditional forward-facing fiber, and it only emits light forward toward the target; the other type of that adopts the side-firing fiber, which is capable of redirecting light laterally at different angles.^1^^,^^4^ Compared with the former, the latter may overcome some accessibility limitation to the treatment of such as the larynx, which is thus of great value to be developed.^1^^,^^4^

Polishing the fiber tip with a certain bevel angle is an important and frequently used way to produce side-firing fibers.^5^^,^^6^ When this fiber is adopted in the instrument, the optical field characteristic at the side-firing emission port, especially in the axial direction, is significantly related to the bevel angle,^7^ which would be the key factor that affects the final effect of the laser treatment.^4^^,^^7^ Because of that, acquiring the quantitative optical field characteristics under the different bevel angles would be in favor of the surgery precision. In 2010, A. Karpiouk et al. analyzed the total internal reflection characteristics on the fiber’s beveled end face, which affects the transmission efficiency, and the analytic expressions for the two crucial bevel angles are obtained, which could be used for distinguishing the different total internal reflection situations.^8^ In 2019, M. Basij et al. analyzed the reflection angle characteristics on the fiber’s beveled end face, and the analytic relationship between the reflection angles and the bevel angle is obtained.^7^ Besides the research based on the analytic expression, the optical field distribution analysis using the commercial optical simulation softwares, which may mainly use the ray tracing algorithm, was also done, and it was generally conducted under a certain bevel angle, such as the angle of 40 degree or 45 degree.^9^^,^^10^ These research results have offered some important reference to the side-firing optical field characteristics.

However, the optical field characteristics should be further researched, which would make the instrument with the side-firing fiber be better utilized in the precision medicine. Firstly, the bevel angle could continuously vary from larger than 0 to less than π/2 rad in theory, and in such a wide variation range, the entire optical path, which may pass through such as the interface between the silica cap and the air,^3^^,^^11^ could significantly change, which would substantially change the emitted beam’s characteristics. Obviously, mastering this optical field characteristic would be conducive to the instrument’s precise design, required for the different practical applications; however, the quantitative research of such characteristic, which covers the entire range of the bevel angle, has not been done in detail in the current reported research. Secondly, the side-firing tip is generally covered by some special protective machine elements, such as the metal cap with the emission opening.^11^^,^^12^^,^^13^^,^^14^ The compatibility between the optical field range and the machine element’s geometric dimension is one of the most important factors that should be considered, however, the in-depth quantitative study for that has not been done.

The research of this paper aims at the laser instrument with the side-firing fiber, whose emission end is covered by a silica cap and a metal cap with an emission opening.^11^ The characteristics of the emitted beam’s longitudinal optical field are theoretically explored in detail, which is considered in the entire continuous range of the bevel angle. The analysis is mainly based on the analytical expressions for the main characteristic parameters, such as the emitted beam’s divergence angle and coverage. Because of that, the characteristic parameters could be directly obtained by the mathematical expressions within the continuous variation range of the bevel angle. Compared with the analysis method based on the ray tracing algorithm simulation software,^9^^,^^10^ which generally uses the numerical calculations to acquire the optical characteristics under each discrete bevel angle, the method in this paper would be more applicable to discuss the characteristics in the entire continuous range of the bevel angle. The different characteristics of the internal optical path and the side-firing emission angle are analyzed, which is under the different subdivided ranges of the complete bevel angle range. And how the bevel angle affects the emitted beam’s divergence angle and coverage is discussed. Based on that, the size requirement for the emission opening’s edge is studied. The motivation of this paper’s study is to provide a quantitative theoretical basis for the optimization design of this laser instrument.

## Results

### Structure and optical path

**Figure 1:**
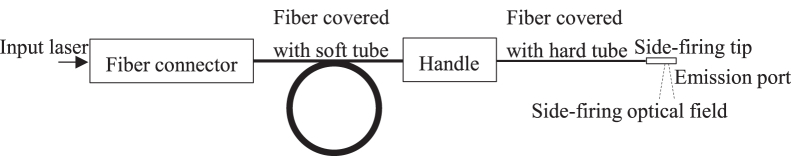
Basic instrument structure

Figure 1 is the basic structure of the instrument, which is generally adopted by the manufacturers.^11^^,^^13^^,^^14^ The instrument consists of five parts, which are the fiber connector, the fiber covered with the soft tube, the handle, the fiber covered with the hard tube and the side-firing tip with the emission port successively. The multimode silica fiber is used in the instrument, which is easier to be launched with optical power than the single-mode silica fiber.^2^^,^^11^^,^^13^^,^^15^ Accordingly, the theoretical analysis in this paper is for a multimode silica fiber. The used fiber is of low OH^−^, because of that, the wavelength ***λ*** of the used laser could be from about 500 nm to 2300 nm, such as 532 nm, 630 nm, 1.44 μm, and 2.12 μm.^1^^,^^11^^,^^12^^,^^13^^,^^15^^,^^16^ The laser is input into the instrument through the fiber connector, and it is finally output at the side-firing emission port. The analysis of this paper concentrates on the characteristics of the side-firing emission optical field.

**Figure 2:**
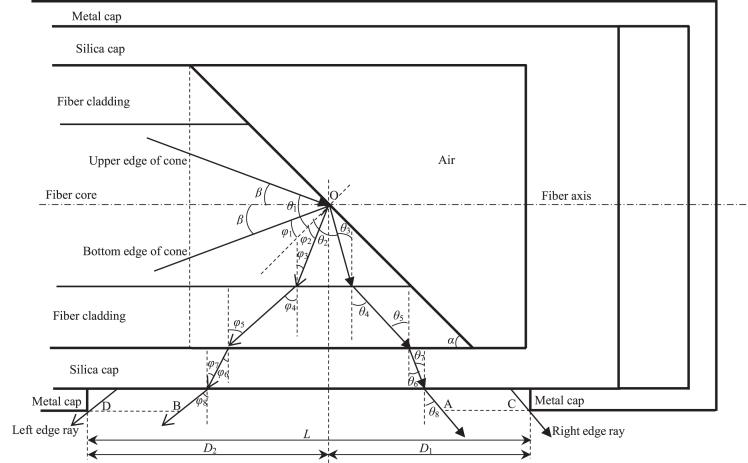
Axial section of side-firing tip

Figure 2 is the axial section of the side-firing tip, which shows the internal structure and optical path.^11^ The side-firing tip mainly consists of three parts, which are the fiber, the silica cap and the metal cap.^11^^,^^13^ The fiber’s end face is polished to make the angle between the end face and the fiber axis be less than π/2 rad, which is the bevel angle *α*.^5^^,^^7^ The fiber’s end is encapsulated into the silica cap, which is transparent and inserted into the metal cap with an emission opening, and to simplify the analysis, it is assumed that the diameter of the fiber cladding is equal to the inner diameter of the silica cap and the outer diameter of the silica cap is equal to the inner diameter of the metal cap.

In this paper, the method of beam deflection is to make the rays, coming through the fiber, reflected on the fiber’s beveled end face.^13^^,^^14^ Because of that, the silica cap is air-filled to preserve an air environment, which would maintain the air/silica interface for total internal reflection.^6^^,^^13^^,^^14^^,^^17^ The adopting of the silica cap would induce some additional losses for the fiber’s side-firing emission, which is due to the following two reasons.^3^ Firstly, the cap’s silica material itself would induce some loss, which could be calculated by multiplying the silica cap wall’s thickness by the attenuation for silica; secondly, at each interface, such as the interface between the fiber and the silica cap, the Fresnel losses would be induced.^3^

The metal cap, which is not utilized as a reflector in this paper, is mainly used for mechanical strength, shielding any scattered light from escaping and reflecting the laser energy reflected back from the target tissue.^11^^,^^13^^,^^14^ The metal cap could be composed of gold, silver, copper, or aluminum.^11^ For a certain wavelength of the laser, the reflectivity varies with different metals. For the metals of copper, aluminum, gold, and silver, when the laser’s wavelength is about 500 nm, the approximate reflectivities are 44%, 91%, 42%, and 95%, respectively, and when the wavelength is about 700 nm, the approximate reflectivities are 83%, 89%, 93%, and 96%, respectively.^18^^,^^19^ In general, the silver always has the largest reflectivity. On the other side, for a certain metal, the reflectivity increases as the wavelength increases.^18^^,^^19^ Taking silver as an example, the reflectivity would increase from about 95% to 98.6% as the wavelength increases from about 500 nm to 10600 nm.^18^^,^^19^ Therefore, when a silver metal cap or a long wavelength is selected, the inner surface of the metal cap would reflect more internal scattered light, which may finally be included by the emitted beam, and the outer surface of that would reflect more laser energy from the target tissue.^11^^,^^13^^,^^14^ Besides the reflectivity, the skin depth also varies as the metal and the wavelength change. For a certain wavelength, the skin depth increases in an order from silver, copper, gold to aluminum; on the other hand, for a certain metal, the skin depth increases as the wavelength increases.^20^ Therefore, in theory, selecting the silver cap together with the laser of short wavelength is conducive for shielding the scattered light.^11^^,^^13^^,^^14^^,^^21^ However, under the wavelength from 500 nm to 2300 nm, for any of the four metals, the skin depth would be much less than the thickness of the metal cap’s tube wall,^20^ which may be from 10 μm to 1000 μm.^11^ Because of that, in practice, any of the metals is adequate for shielding the laser with the considered wavelength.

The selected axial section shown in Figure 2 is coincident with the fiber’s meridian plane, perpendicular to the beveled end face, since the maximum range of the field distribution along the fiber axis, which is looked into in this paper, is mainly related to the meridional rays in this meridian plane.^9^^,^^12^ The rays inside the fiber could propagate within the certain cone, which has the upper and bottom edges.^8^ When the two edge rays are incident into the end face, they would go through the different optical paths, respectively, and finally they may emit from the emission opening.^7^ Here, *β* is the angle between the cone’s edge and the fiber axis; *θ*_1_ to *θ*_8_ are the incident, reflection or refraction angles corresponding to the upper edge ray’s optical path, and the reflection and refraction angles could be obtained according to reflection law and Snell’s law, respectively;^22^^,^^23^ *φ*_1_ to *φ*_8_ are the incident, reflection or refraction angles corresponding to the bottom edge ray’s optical path; O is the intersection point of the fiber axis and the beveled end face; A and B are the intersection points of the emitted rays and the outer surface of the metal cap, which, respectively, originates from the upper and bottom edge rays with the same incident point on the beveled end face; the right edge ray is the emitted ray originating from the upper edge ray, whose incident point is at the bottom edge of the beveled end face within the fiber core, and C is the intersection point of this ray and the outer surface of the metal cap; the left edge ray is the emitted ray originating from the bottom edge ray, whose incident point is at the upper edge of the beveled end face within the fiber core, and D is the intersection point of this ray and the outer surface of the metal cap; *L* is the distance between the left and right edges of the emitted beam’s optical field, which is distributed on the outer surface of the metal cap; *D*_1_ is the crucial distance between the right edge of the opening and O, which is in the direction of the fiber axis; *D*_2_ is the crucial distance between the left edge of the opening and O, which is in the direction of the fiber axis.

Since the three-dimensional internal structure of the side-firing tip is symmetric with respect to the selected meridian plane, shown in Figure 2, the intensity distribution of the emitted beam would be symmetric with respect to this plane.^9^^,^^11^^,^^12^^,^^13^^,^^14^ Supposing the target tissue surface is parallel to the fiber axis, which is very close to the outer surface of the metal cap, the spot, projected on the tissue surface, may have a symmetric elliptical shape in general, and its length, in the direction of the fiber axis, increases as the distance *L* increases.^12^^,^^13^^,^^14^^,^^16^

### Crucial bevel angle

According to *α*′s different ranges, corresponding to the different optical path characteristics, the ten crucial angles could be summarized in Table 1, which will be further discussed in the following section. Here, *n*_1_ and *n*_2_ are the refractive indices of the fiber core and cladding, respectively; *θ*_c1_ is the crucial angle of the interface between the fiber core and cladding.

**Table 1:** Crucial angles for α

Symbol	Expression	Symbol	Expression
*α*_0_	*β*	*α*_5_	π/2-arcsin(*n*_2_/*n*_1_)/2
*α*_1_	arcsin(-1/*n*_1_)/2+arcsin(*n*_2_/*n*_1_)/2	*α*_6_	arcsin(1/*n*_1_)/2+arcsin(*n*_2_/*n*_1_)/2
*α*_2_	π/2-arcsin(1/*n*_1_)/2-arcsin(*n*_2_/*n*_1_)/2	*α*_7_	π-arcsin(*n*_2_/*n*_1_)-arcsin(1/*n*_1_)
*α*_3_	arcsin(*n*_2_/*n*_1_)-arcsin(1/*n*_1_)	*α*_8_	π/2+arcsin(1/*n*_1_)/2-arcsin(*n*_2_/*n*_1_)/2
*α*_4_	arcsin(*n*_2_/*n*_1_)/2	*α*_9_	*θ*_c1_

## Discussion

### Characteristics of crucial bevel angle

**Figure 3:**
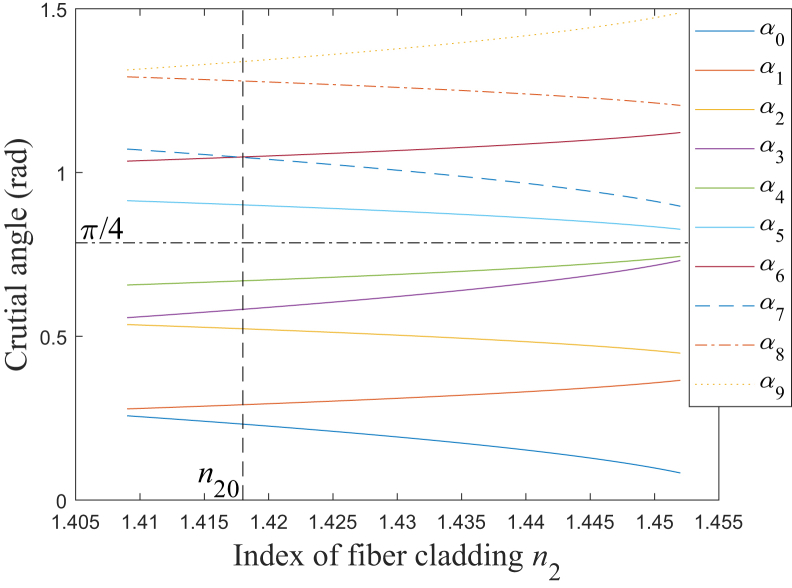
Relationship between crucial angles and *n*_2_ *α*_0_: blue solid line; *α*_1_: red solid line; *α*_2_: yellow solid line; *α*_3_: purple solid line; *α*_4_: green solid line; *α*_5_: cyan solid line; *α*_6_: brown solid line; *α*_7_: blue dashed line; *α*_8_: red dash-dotted line; *α*_9_: yellow dotted line.

In Table 1, the ten crucial angles for *α* have been summarized. Obviously, these crucial angles vary with the refractive indices *n*_1_ and *n*_2_. Figure 3 is the simulation results for the relationship between the crucial angles and *n*_2_. The fiber core refractive index *n*_1_ is set as 1.457, corresponding to the laser’s wavelength being around 640 nm, and the fiber cladding refractive index *n*_2_ is set from 1.409 to 1.452, which corresponds to the fiber’s numerical aperture (NA) ranging from about 0.37 to 0.12.^10^^,^^15^^,^^24^^,^^25^^,^^26^^,^^27^^,^^28^^,^^29^^,^^30^ The value of NA has an important impact on the light propagation.^15^ Firstly, at the input end of the fiber, the light acceptance capability increases as NA increases, which means that the source-to-fiber optical power coupling efficiency increases as NA increases.^15^ Because of that, the emitted power at the emission port may generally increase as NA increases. Secondly, the number of modes, which the multimode fiber can support, increases as NA increases, which means that the modal dispersion may increase with NA.^15^^,^^31^ Because of that, for the pulsed laser, as NA increases, the width and peak power of the emitted laser pulse may increase and decrease, respectively.^12^^,^^31^ Besides these effects, NA would also affect the characteristics of emitted beam’s divergence angle and coverage, which would be described in detail in the following paragraphs.

Based on the relationship among these crucial angles, to make all the rays in the cone be side-firing emitted, *α* should be in the range of *α*_2_<*α*<*α*_6_, and it is found that *α*_2_ decreases and *α*_6_ increases as *n*_2_ increases, which means that decreasing NA is advantageous for increasing the range of *α*. The crucial angle *α*_3_ increases as *n*_2_ increases, which means that decreasing NA is also advantageous for increasing the range of *α* to make all the rays achieve the total internal reflection on the beveled end face.

In the range of *α*_2_<*α*<*α*_6_, there exists different situations corresponding to different subdivided ranges. When *n*_2_ is in the range of *n*_2_ ≤ 1.418 and *n*_2_ > 1.418, which corresponds to the different two ranges for NA, there exists 6 and 7 different situations, respectively, which is shown in Table 2. When *n*_2_ is not larger than 1.418, there always exists some rays of total internal reflection on the beveled end face. Nevertheless, when *n*_2_ is larger than 1.418, once *α* increases to be larger than *α*_7_, there is no ray of total internal reflection, which would seriously decrease the transmission efficiency and should be avoided. Although coating the end surface may solve this problem, it would increase the complexity and the cost of the fabrication process.^9^^,^^32^

**Table 2:** Different situations with different ranges of *α*

Range of *α*		Quantity of ray totally internally reflected on beveled end face	Inclination direction of emitted upper edge ray	Inclination direction of emitted bottom edge ray
*n*_2_ ≤ 1.418	*n*_2_ > 1.418			
*α*_2_<*α*≤*α*_3_		All	Right side of normal line	Right side of normal line
*α*_3_<*α*<*α*_4_		Partial	Right side of normal line	Right side of normal line
*α* = *α*_4_		Partial	Right side of normal line	Perpendicular to interface
*α*_4_<*α*<*α*_5_		Partial	Right side of normal line	Left side of normal line
*α* = *α*_5_		Partial	Perpendicular to interface	Left side of normal line
*α*_5_<*α*<*α*_6_	*α*_5_<*α*≤*α*_7_	Partial	Left side of normal line	Left side of normal line
*/*	*α*_7_<*α*<*α*_6_	None	Left side of normal line	Left side of normal line

In practice, the requirements for the emitted rays’ general emission angle may be different under the different application scenarios.^1^^,^^4^^,^^13^^,^^14^ Obviously, the emission angle of each emitted ray is between the emission angles of the two special emitted rays, originating from the upper and bottom edge rays, respectively. Because of that, according to the results in Table 2, the different general emission angle of the emitted beam could be definitely realized by selecting the different suitable ranges of *α*. When *α* is designed to be in the range of *α*_2_<*α*<*α*_4_, all the rays would be emitted toward the right side of their respective normal lines, and in this case, the general emitted beam would incline to the direction of approaching the instrument’s distal end; in particular, for the purpose to increase the transmission efficiency, sometimes *α* may be designed to be in the range of *α*_2_<*α*≤*α*_3_ to make all the rays in the cone achieve the total internal reflection,^5^^,^^12^^,^^13^^,^^14^ and in this case, the general emitted beam would always have the aforementioned inclination directions. On the other hand, when *α* is designed to be in the range of *α*_5_<*α*<*α*_6_, all the rays would be emitted toward the left side of their respective normal lines, thus, the general emitted beam would incline to the direction of being away from the instrument’s distal end. In addition, when *α* is designed to be in the range of *α*_4_<*α*<*α*_5_, the different rays would be emitted toward both the left and right sides of their respective normal lines, and this means that the general emitted beam would be separated into two parts, which incline to the two directions of approaching and being away from the instrument’s distal end, respectively; particularly, when *α* is equal to π/4 rad, which is also included in the range of *α*_4_<*α*<*α*_5_ as shown in Figure 3, the general emitted beam would be symmetrically distributed in the aforementioned two inclination directions.^3^^,^^5^^,^^9^^,^^32^

According to Fresnel’s equation, the polarization states of the emitted rays would be affected by α.^2^^,^^15^^,^^22^^,^^23^ On the one side, the two reflectivities for parallel and perpendicular polarizations, respectively, may be different when the rays are incident into the beveled end face, and the difference between the two reflectivities may vary with the incident angle, which is decided by α. On the other side, the two transmissivities for parallel and perpendicular polarizations, respectively, may be different when the rays pass through each interfaces, and the difference between the two transmissivities may vary with the incident angle, which is decided by α. However, the used multimode silica fiber is not polarization maintaining.^15^ That means the polarization state would randomly vary with time when the laser is transmitted through the whole fiber. Because of that, in the practical application, the polarization state of the final emitted beam would be random under a certain α.

For the commercial product of this instrument, the exit angle, the divergence and the transmission efficiency are three of the most important parameters, and each manufacturer’s product has its definite values for these parameters.^13^^,^^14^ The certain exit angle and divergence correspond to the certain inclination directions of the emitted upper and bottom edge rays; on the other side, the transmission efficiency is related to the total internal reflection situation. Because of that, in the practical design process for the manufacturers, based on these analysis results, the range of the bevel angle could be initially determined for the required edge rays’ inclination directions and total internal reflection situation, which is conducive for the further fine design.

### Characteristics of emitted beam’s divergence angle

**Figure 4:**
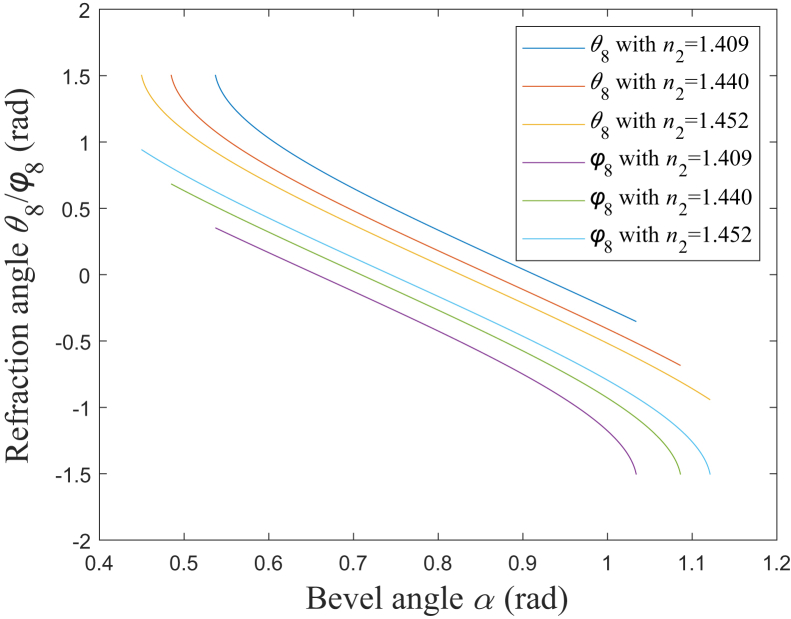
Relationship between *θ*_8_/*φ*_8_ and *α* *θ*_8_ with *n*_2_ = 1.409: blue solid line; *θ*_8_ with *n*_2_ = 1.440: red solid line; *θ*_8_ with *n*_2_ = 1.452: yellow solid line; *φ*_8_ with *n*_2_ = 1.409: purple solid line; *φ*_8_ with *n*_2_ = 1.440: green solid line; *φ*_8_ with *n*_2_ = 1.452: cyan solid line.

In Figure 2, *θ*_8_ and *φ*_8_ are the refraction angles of the interface between the silica cap and the air, which correspond to the upper and bottom edge rays, respectively. According to Equations 10 and 13, Figure 4 shows the simulation results of the relationship between *θ*_8_/*φ*_8_ and *α*. The range of *α* is set to be from *α*_2_+*δ* to *α*_6_-*δ* with *δ* being 10^−3^ rad, which satisfies *α*_2_<*α*<*α*_6_; and the fiber cladding refractive index *n*_2_ is set as 1.409, 1.440 and 1.452, which correspond to NA being about 0.37, 0.22 and 0.12, respectively.^10^^,^^15^^,^^27^^,^^28^^,^^29^^,^^30^ It is found that both *θ*_8_ and *φ*_8_ generally increases as NA increases. For a certain NA, both *θ*_8_ and *φ*_8_ decrease as *α* increases. Particularly, *θ*_8_ decreases sharply when *α* is less than a certain value, and *φ*_8_ decreases sharply when *α* is larger than a certain value. Besides, according to Equations 10 and 13, when *α* is π/4 rad, *θ*_8_ would be equal to -*φ*_8_, which corresponds to the axisymmetric emitted beam.^3^^,^^5^^,^^9^^,^^32^

**Figure 5:**
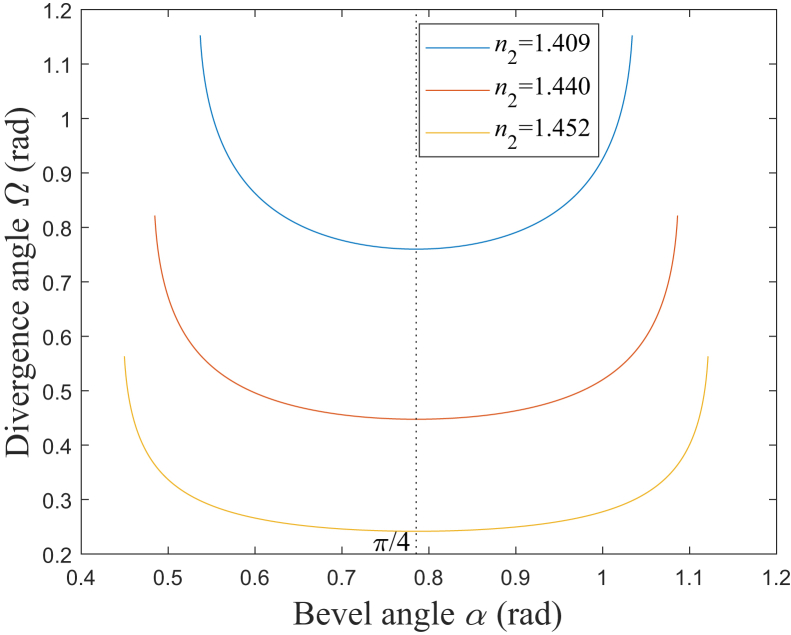
Relationship between *Ω* and *α* *n*_2_ = 1.409: blue solid line; *n*_2_ = 1.440: red solid line; *n*_2_ = 1.452: yellow solid line.

The divergence angle of the emitted beam, in the fiber axis direction, is defined as *Ω*.^12^ According to Equation 14, Figure 5 shows the simulation results of the relationship between *Ω* and *α*. It could be found that *Ω* generally increases as NA increases. For a certain NA, when *α* is π/4 rad, which corresponds to the extreme point of *Ω* according to Equation 15, *Ω* reaches its minimum, which is given by NA,^12^ and this minimum is *Ω*_0_, described in Equation 16. As *α* deviates from π/4 rad, *Ω* increases, and particularly, when *α* is less or larger than a certain value, *Ω* increases sharply. Therefore, in order to limit the value of *Ω* to avoid the serious diffusion, *α* should be controlled to be within a certain range.

**Figure 6:**
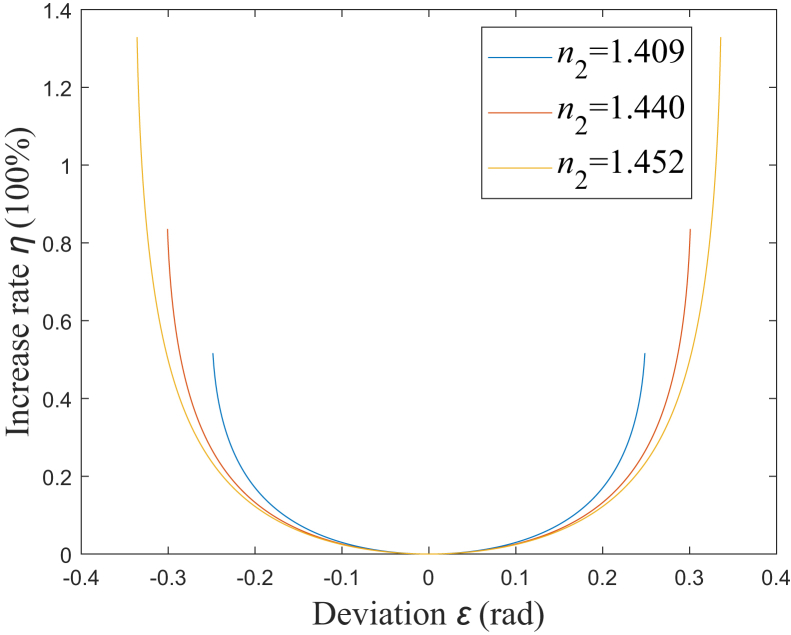
Relationship between *η* and *ε* *n*_2_ = 1.409: blue solid line; *n*_2_ = 1.440: red solid line; *n*_2_ = 1.452: yellow solid line.

When *α* deviates from π/4 rad for *ε*, the increase rate for *Ω* is defined as *η*. According to Equation 27, Figure 6 shows the simulation results of the relationship between *η* and *ε*. It could be found that for a larger NA, *η* would increase faster as |*ε*| increases. Here, *η* is assumed to be limited to being less than *η*_t_. When *η*_t_ is set as 5%, |*ε*| should be less than about 0.1258, 0.1350, and 0.1385, which corresponds to NA being 0.37, 0.22, and 0.12, respectively. Therefore, a smaller NA would correspond to a larger range of *α*, in which the rate of increase for *Ω* is limited to a certain range. When *α* is set to be *α*_3_, which are 0.5570 rad, 0.6614 rad, and 0.7315 rad corresponding to NA being 0.37, 0.22, and 0.12, respectively, *η* would be about 28.29%, 4.12%, and 0.68%, respectively. Therefore, when the total internal reflection on the beveled end face occurs for all the rays, *η* could be less than 5% with NA being 0.22 or 0.12, however, *η* would increase to be larger than 5% with NA being 0.37.

As mentioned previously, the divergence is one of the most important parameters for the commercial product of this instrument.^13^^,^^14^ In the practical design process for the manufacturers, these analysis results could help to precisely control the divergence and provide the theoretical basis for evaluating the tolerance.

### Characteristics of emitted beam’s coverage

**Figure 7:**
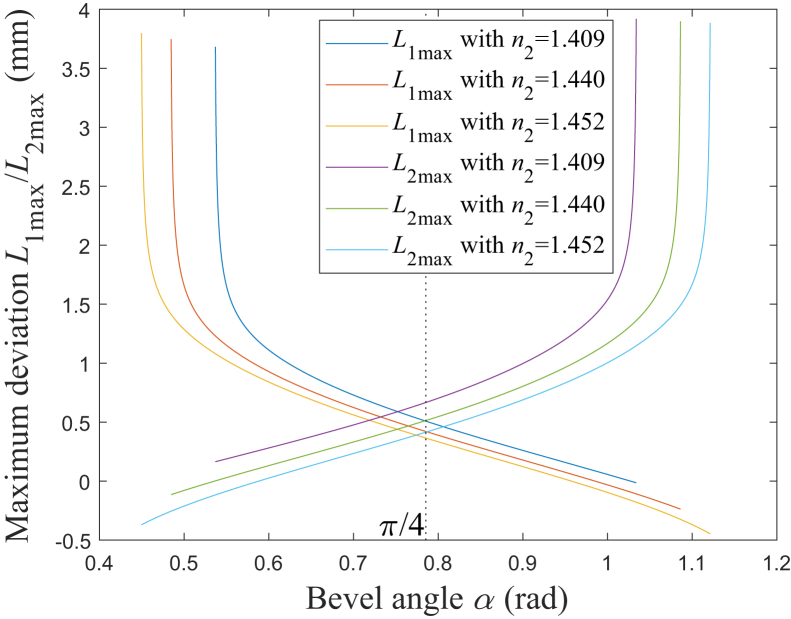
Relationship between *L*_1max_/*L*_2max_ and *α* *L*_1max_ with *n*_2_ = 1.409: blue solid line; *L*_1max_ with *n*_2_ = 1.440: red solid line; *L*_1max_ with *n*_2_ = 1.452: yellow solid line; *L*_2max_ with *n*_2_ = 1.409: purple solid line; *L*_2max_ with *n*_2_ = 1.440: green solid line; *L*_2max_ with *n*_2_ = 1.452: cyan solid line.

In the direction of the fiber axis, the deviation for A from O is defined as *L*_1_, and the deviation for B from O is defined as *L*_2_. The maxima of *L*_1_ and *L*_2_ are defined as *L*_1max_ and *L*_2max_, respectively. According to Equations 20 and 23, Figure 7 shows the simulation results of the relationship between *L*_1max_/*L*_2max_ and *α*. The refractive index of the silica cap *n*_3_ is set as 1.457, corresponding to the laser’s wavelength being around 640 nm, the diameter of the fiber core *d*_1_ is set as 0.6 mm, the diameter of the fiber cladding *d*_2_ is set as 0.66 mm,^24^^,^^25^^,^^26^^,^^28^^,^^29^^,^^30^ the outer diameter of the silica cap *d*_3_ is set as 1.65 mm, and the outer diameter of the metal cap *d*_4_ is set as 2 mm. It is found that both *L*_1max_ and *L*_2max_ generally increases as NA increases. When *α* is at a certain value *α*_e_, which is close to π/4 rad and increases as NA decreases, *L*_1max_ is equal to *L*_2max_. For a certain NA, *L*_1max_ decreases and *L*_2max_ increases as *α* increases. In particular, when *α* is less than a certain value, *L*_1max_ decreases sharply; on the other side, when *α* is larger than a certain value, *L*_2max_ increases sharply.

**Figure 8:**
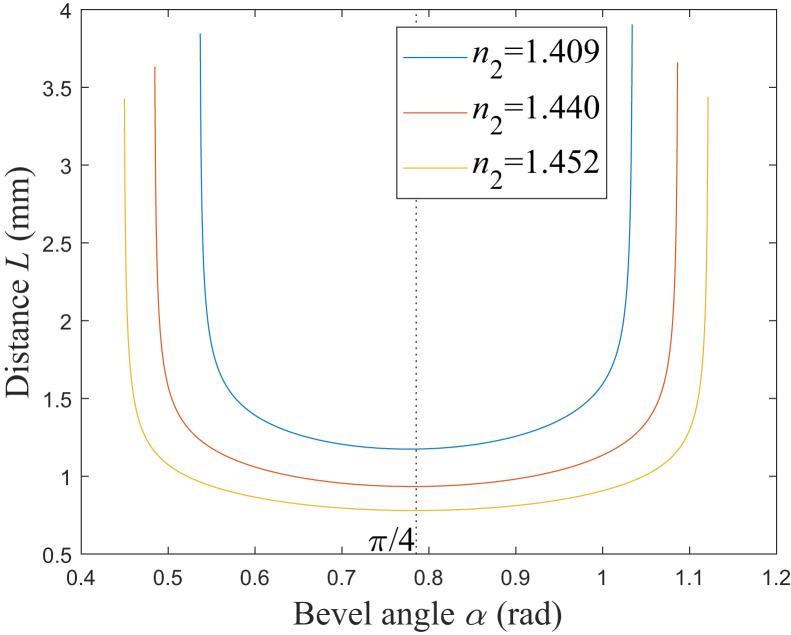
Relationship between *L* and *α* *n*_2_ = 1.409: blue solid line; *n*_2_ = 1.440: red solid line; *n*_2_ = 1.452: yellow solid line.

According to Equation 24, Figure 8 is the simulation results of the relationship between *L* and *α*. It is found that *L* generally increases as NA increases. When *α* is at a certain value *α*_m_, which is close to π/4 rad, *L* reaches its minimum. According to the numerical simulation results, *α*_m_ are 0.7771 rad, 0.7809 rad, and 0.7835 rad, which correspond to NA being 0.37, 0.22, and 0.12, and it could be found that *α*_m_ increases as NA decreases. For a certain NA, when *α* is less than *α*_m_, *L* decreases as *α* increases, and particularly, when *α* is less than a certain value, *L* decreases sharply; on the other hand, when *α* is larger than *α*_m_, *L* increases as *α* increases, and in particular, when *α* is larger than a certain value, *L* increases sharply.

According to Figure 8, the spot’s length, in the direction of the fiber axis, would vary with *α*, which increases as *L* increases, mentioned previously. On the other side, the spot’s length, in the vertical direction of the fiber axis, may be not so sensitive to *α* as that in the direction of the fiber axis. The reason is that the variation of *α* would mainly lead to the variation of the rays’ reflection angles, in the direction of the fiber axis but not the vertical direction, and the emitted beam’s coverage, in the vertical direction of the fiber axis, may be not so sensitive to *α* as that in the direction of the fiber axis.^5^^,^^6^^,^^7^^,^^8^^,^^9^ Therefore, the spot’s length, in the vertical direction, is assumed to be constant when *α* varies in this paper. Under a certain emitted power, the intensity of the emitted beam generally decreases as the spot’s length increases, which causes the spot size to increase.^14^^,^^33^^,^^34^ Generally, to increase the transmission efficiency, *α* is designed to be in the range of *α*_2_<*α*≤*α*_3_, in which all the rays are totally internally reflected on the beveled end face.^5^^,^^12^^,^^13^^,^^14^ Here, the crucial angle *α*_2_ are 0.5359 rad, 0.4836 rad, and 0.4486 rad, which correspond to NA being 0.37, 0.22, and 0.12, respectively; the crucial angle *α*_3_ are 0.5570 rad, 0.6614 rad, and 0.7315 rad, which correspond to NA being 0.37, 0.22, and 0.12, respectively. When the Fresnel losses are neglected,^3^^,^^23^ the emitted power may be approximately assumed to be constant with the variable *α*. According to the characteristic of *L*, shown in Figure 8, when *α* is in the range of *α*_2_<*α*≤*α*_3_, the intensity of the emitted beam generally trends to decrease as *α* decreases, and particularly, when *α* decreases to be less than a certain value, the intensity decreases sharply.

**Figure 9:**
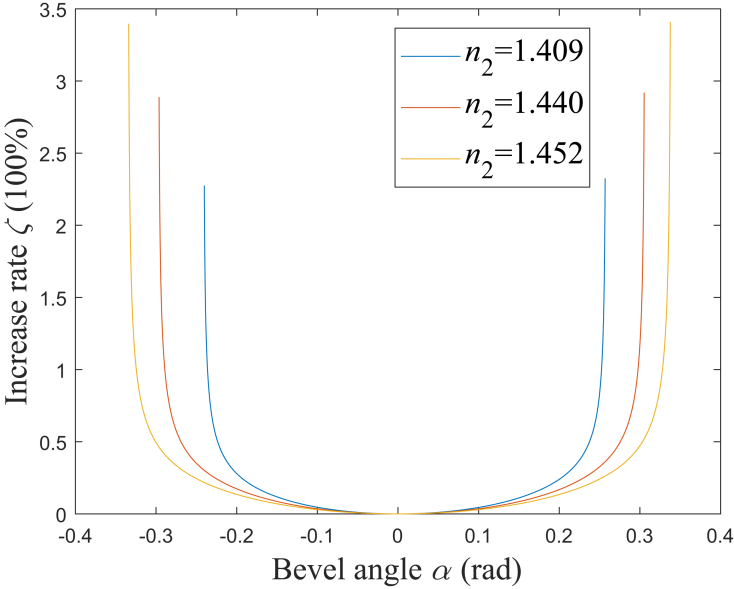
Relationship between *ζ* and *ε* *n*_2_ = 1.409: blue solid line; *n*_2_ = 1.440: red solid line; *n*_2_ = 1.452: yellow solid line.

When *α* deviates from *α*_m_ for *ε*, the increase rate for *L* is defined as *ζ*. According to Equation 30, Figure 9 shows the simulation results of the relationship between *ζ* and *ε*. It could be found that for a larger NA, *ζ* would increase faster as *ε* deviates from 0. Here, *ζ* is assumed to be limited to being less than *ζ*_t_. When *ζ*_t_ is set as 5%, the range of *ε* should be −0.1036<*ε*<0.1051, −0.1171<*ε*<0.1176, and −0.1284<*ε*<0.1285, which corresponds to NA being 0.37, 0.22, and 0.12, respectively. Therefore, a smaller NA would correspond to a larger range of *α*, in which the rate of increase for *L* is limited to a certain range. When *α* is set to be *α*_3_, *ζ* would be 44.74%, 5.22%, and 0.78%, corresponding to NA being 0.37, 0.22, and 0.12, respectively. Therefore, when all the rays are totally internally reflected on the beveled end, *ζ* could be limited to being less than 5% with NA being 0.12, however, *ζ* would increase to be larger than 5% with NA being 0.37 or 0.22.

**Figure 10:**
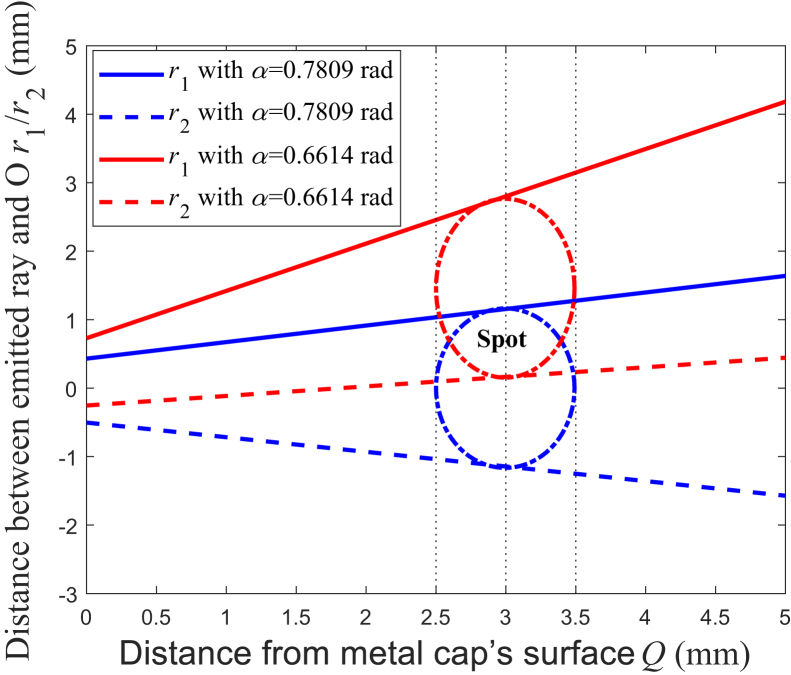
Emitted beam trajectories *r*_1_ with *α* = 0.7809 rad: blue solid line; *r*_2_ with *α* = 0.7809 rad: blue dashed line; *r*_1_ with *α* = 0.6614 rad: red solid line; *r*_2_ with *α* = 0.6614 rad: red dashed line.

According to Equations 31 and 32, Figure 10 is the simulation results of the two emitted beam trajectories, and each of them consists of the emitted rays originating from the upper and bottom edge rays.^9^^,^^12^^,^^14^^,^^23^ The fiber cladding refractive index *n*_2_ is set as 1.440, which corresponds to NA being about 0.22. For the beam trajectory with blue color, *α* is set as *α*_m_, which is 0.7809 rad, and in this case, *L* would be at its minimum according to the results mentioned previously. For the beam trajectory with red color, *α* is set as *α*_3_, which is 0.6614 rad, and in this case, the total internal reflection on the beveled end face occurs for all the rays. The blue beam has the two parts, which incline to the two directions of approaching and being away from the instrument’s distal end, respectively; however, the red beam just incline to the direction of approaching the distal end. The beam trajectories would reflect the spot shape under different conditions. To explain how the length of the spot varies with *α,* which is in the direction of the fiber axis, the schematics of the two spots are shown at the same distance from the metal cap’s surface, which is 3 mm. The spot with blue color has the minimum length in the direction of the fiber axis. When *α* decreases to *α*_3_, this spot is lengthened to the spot with red color. For the spots, under the condition of all the rays being totally internally reflected, the red spot has the minimum length, in the direction of the fiber axis.

For the commercial product of this instrument, the spot size is one of the most important parameters, and each manufacturer’s product has its definite value for this parameter.^13^^,^^14^ The certain spot size corresponds to the certain emitted beam’s coverage. Because of that, in the practical design process for the manufacturers, these analysis results could help to precisely control the spot size and provide the theoretical basis for evaluating the tolerance.

### Characteristics of emission opening’s required size

**Figure 11:**
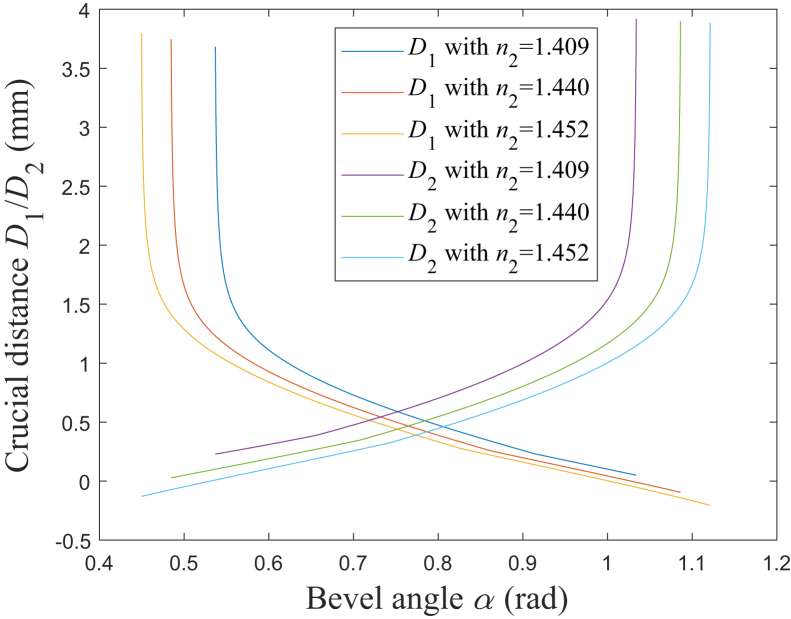
Relationship between *D*_1_/*D*_2_ and *α* *D*_1_ with *n*_2_ = 1.409: blue solid line; *D*_1_ with *n*_2_ = 1.440: red solid line; *D*_1_ with *n*_2_ = 1.452: yellow solid line; *D*_2_ with *n*_2_ = 1.409: purple solid line; *D*_2_ with *n*_2_ = 1.440: green solid line; *D*_2_ with *n*_2_ = 1.452: cyan solid line.

According to Equations 33 and 34, Figure 11 shows the simulation results of the relationship between *D*_1_/*D*_2_ and *α*. It could be found that *D*_1_ decreases and *D*_2_ increases as *α* increases. Compared with the simulation results in Figure 7, it is found that the general variation characteristic of *D*_1_/*D*_2_ is similar to that of *L*_1max_/*L*_2max_, and both the corresponding two curves change sharply before or after a same value for *α*. Nevertheless, *D*_1_ would be slightly larger than *L*_1max_ when *α* is larger than *α*_5_ together with *D*_2_ being slightly larger than *L*_2max_ when *α* is less than *α*_4_, which could be obtained by Equations 33 and 34.

In practice, there exists the machining tolerance for the bevel angle *α*.^22^ Here, the plus and minus tolerances for *α* are defined as +*σ* and –*υ*, respectively. Considering the machining tolerance, to ensure no emitted ray being blocked, the actual crucial distance between the right and left edges of the emission opening is defined as *D*’.

**Figure 12:**
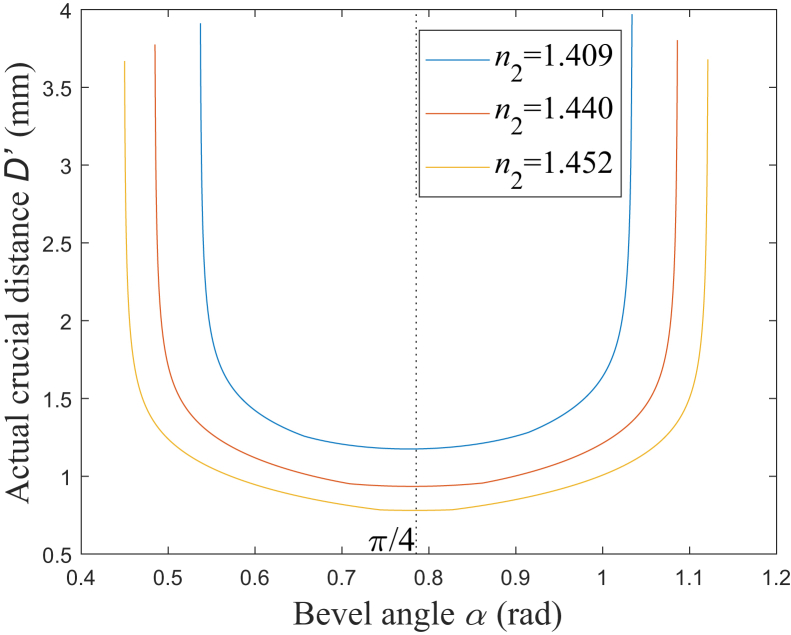
Relationship between *D*′ and *α* *n*_2_ = 1.409: blue solid line; *n*_2_ = 1.440: red solid line; *n*_2_ = 1.452: yellow solid line.

According to Equation 37, Figure 12 shows the simulation results of the relationship between *D*′ and *α*. The plus and minus tolerances +*σ* and –*υ* are set to be +2 × 10^−4^ rad and −2 × 10^−4^ rad, respectively, and the range of *α* is set to be from *α*_2_+*δ*+*υ* to *α*_6_-*δ*-*σ* with *δ* being 10^−3^ rad, which satisfies *α*_2_+*υ*<*α*<*α*_6_-*σ*. Compared with the simulation results in Figure 8, it could be found that the general variation characteristic of *D*′ is similar to that of *L*. There also exists a minimum for *D*′ when *α*′s value is close to π/4 rad, which are 0.7770 rad, 0.7809 rad, and 0.7835 rad corresponding to NA being 0.37, 0.22, and 0.12, respectively. However, *D*′ is slightly larger than *L*, and the curves for *D*′ are obviously steeper than those for *L* when *α* is less than *α*_4_ or larger than *α*_5_, which corresponds to all the emitted rays being toward the same side of the respective normal line.

In the practical situation, some rays, included in such as the secondary beam,^13^^,^^14^ may be reflected back into the fiber by the metal cap, and part of these rays would finally be emitted through the side surface of the fiber. Because of that, the practical emitted beam’s divergence angle and coverage may be larger than the theoretical results in this paper, which just considers the emitted rays originating from the cone. However, most of the enlarged part of the emitted beam may be shielded by the side part of the metal cap, whose designed emission opening just matches the emitted rays originating from the cone.^13^^,^^14^

For the commercial product of this instrument, the metal cap is one of the most important elements, and the size of its emission opening would directly affect the optical characteristics.^11^^,^^13^^,^^14^ In the practical design process for the manufacturers, these analysis results could provide the theoretical basis for the precise design of the emission opening’s size, which would be conducive for the optimization of the optical characteristics finally.

In conclusion, the optical field characteristics of the laser medical instrument with the side-firing fiber are theoretically studied in this paper, and the analysis of that is mainly based on the analytical expressions for the main characteristic parameters. The considered diameters of the fiber core and cladding are 0.6 mm and 0.66 mm, respectively, the fiber core refractive index is 1.457, and the range of the fiber cladding refractive index is from 1.409 to 1.452 corresponding to the NA from 0.37 to 0.12. Table 3 summarizes the range of the relevant parameters considered in this paper. It is found that the complete bevel angle range could be subdivided into multiple sub-ranges by ten crucial angles. These sub-ranges correspond to the different optical path characteristics, respectively. The numerical simulation results indicate that the relationships among these crucial angles are different in NA’s two different ranges, divided by the fiber cladding refractive index being 1.418, and it affects the division for the sub-ranges. There exists a bevel angle range defined by two of the ten crucial angles, in which all the rays in the fiber could achieve the side-firing emission. In that bevel angle range, the emitted beam’s divergence angle reaches its minimum when the bevel angle is at π/4 rad; differing from the divergence, the emitted beam’s coverage achieves its minimum when the bevel angle is at a certain value, which is close to π/4 rad but not π/4 rad. Furthermore, both the divergence angle and coverage increase as the bevel angle deviates from the value being or close to π/4 rad, respectively, for the former or the latter. They increase remarkably when the bevel angle increases or decreases to a certain value. Because of that, it is difficult to make the two parameters limited to a certain value and all the rays achieve total internal reflection on the beveled end face simultaneously. In particular, under a large NA being 0.37, the increase rates for the emitted beam’s divergence angle and coverage would be as large as 28.29% and 44.74%, respectively. Combining the characteristics of the optical field and the emission opening’s structure, the expression for the actual crucial distance between the right and left edges of the emission opening is obtained. The considered refractive index of the silica cap is 1.457, and the outer diameters of the silica and metal caps are 1.65 mm and 2 mm, respectively. The numerical simulation results indicate that this distance has a minimum when the bevel angle is close to π/4 rad but not π/4 rad, such as 0.7809 rad with NA being 0.22, and it increases sharply as the bevel angle increases or decreases to a certain value.

**Table 3:** Range of relevant parameters

Laser wavelength *λ*	Around 640 nm	
Diameter of fiber core *d*_1_	0.6 mm	
Diameter of fiber cladding *d*_2_	0.66 mm	
Outer diameter of silica cap *d*_3_	1.65 mm	
Outer diameter of metal cap *d*_4_	2 mm	
Fiber core refractive index *n*_1_	1.457	
Fiber cladding refractive index *n*_2_	1.409 to 1.452	
Silica cap refractive index *n*_3_	1.457	
Numerical aperture (NA)	0.37 to 0.12	0.37 for *n*_2_ = 1.409
		0.22 for *n*_2_ = 1.440
		0.12 for *n*_2_ = 1.452

The analytical expressions for the concerned characteristic parameters in this paper are mainly based on the edge ray tracing analysis. Therefore, in theory, the research results would be the same as that using the general ray tracing simulation software, whose rationale is also geometrical optics. However, the optical characteristic parameters could be directly obtained by the mathematical expressions within the continuous variation range of the bevel angle; therefore, the method in this paper would be more applicable to achieve the optical characteristics in the entire range of the bevel angle, which would be more convenient for the optimum design especially for the bevel angle.

The different bevel angles in the entire angle range correspond to different optical characteristics, in the direction of the fiber axis. In the practical applications, the different optical characteristics may make the laser reach the different disease, which satisfies different requirements of the laser treatment. The research results of this paper could provide the significant theoretic basis for the further study of the side-firing laser medical instrument.

### Limitations of the study

Further research is needed, including three aspects. Firstly, due to limited conditions, the theoretical results acquired in this paper have not been compared with the experimental characterization of this instrument; therefore, to verify the current results, the experimental research is necessary to be performed in the future. Secondly, the internal scattered light, such as the light reflected back from the internal surface of the metal cap, would influence the characteristics of the emitted beam, which has not been analyzed in detail; because of that, in the entire range of the bevel angle, the quantitative research of the scattered light should be carried out in the next step. Thirdly, the optical characteristics are mainly researched in the direction of the fiber axis in this paper; however, to more comprehensively acquire the characteristics of the emitted beam, in the entire range of the bevel angle, the three-dimensional optical characteristics are needed to be researched in the future. Fourthly, the analysis method in this paper is geometrical optics, which may have some limitations; therefore, in the further research, it is better to combine this method with the wave optics method, which may acquire more accurate optical characteristics.

## Resource availability

### Lead contact

Further information and requests for resources should be directed to and will be fulfilled by the lead contact, Diqing Ying (dqying@zju.edu.cn).

### Materials availability

This study did not generate new unique materials.

### Data and code availability

• The data reported in this paper will be shared by the lead contact upon reasonable request.
• The code reported in this paper will be shared by the lead contact upon reasonable request.
• Any additional information required to reanalyze the data reported in this paper is available from the lead contact upon reasonable request.

## Acknowledgments

This work was supported by Hangzhou Leichi Science & Technology Company Limited providing the funding (university-enterprise cooperation project number: K横20230512).

## Author contributions

Diqing Ying contributed to the study conception and design, acquired the funding, conducted the investigation, performed the research, conducted the theory analysis and simulation, and wrote the paper.

## Declaration of interests

The author has a patent application related to this work.

## STAR★Methods

### Key resources table

**Table:** 

REAGENT or RESOURCE	SOURCE	IDENTIFIER
**Software and algorithms**		

MATLAB R2021b	MathWorks	https://www.mathworks.com/

### Method details

#### Bevel angle with different optical path

The crucial angle of the interface between the fiber core and cladding could be expressed as:^15^

(Equation 1) $$\theta_{\text{c}1}=\arcsin\left(n_{2}/n_{1}\right)$$

The angle *β* could be expressed as:^15^

(Equation 2) $$\beta =\text{π}/2-\theta_{\text{c}1}=\text{π}/2-\arcsin\left(n_{2}/n_{1}\right)$$

The relationship between *θ*_c1_ and *β* is assumed to be *β*<*θ*_c1_, which agrees with the typical values for *n*_1_ and *n*_2_,^24^ and it means that *β* is in the range of 0<*β*<π/4 and *θ*_c1_ is in the range of π/4<*θ*_c1_<π/2. In this paper, the incident or refractive angle of the ray is positive and negative when the ray turns to its normal at an acute angle clockwise and counter-clockwise, respectively.^35^ Then, according to the geometrical relationship, the incident angle of the interface between the fiber core and the air, which corresponds to the upper edge ray, could be expressed as:

(Equation 3) $$\theta_{1}=\arcsin\left(n_{2}/n_{1}\right)+\alpha -\text{π}$$

When *α* is in the range of 0<*α*≤*β*, according to Equations 2 and 3, *θ*_1_ would be in the range of arcsin(*n*_2_/*n*_1_)-π<*θ*_1_≤-π/2, which means that the upper edge ray could not directly reach the beveled end face. In addition, according to the geometrical relationship, part of the residual rays near the upper edge ray, which could reach the end face and be reflected, may not be able to pass through the interface between the fiber core and cladding for total internal reflection, which decreases the transmission efficiency of the side-firing power. To avoid these problems, the range of *α* should be *β*<*α*<π/2.

According to the geometrical relationship, the incident angle of the interface between the fiber core and cladding, which corresponds to the upper edge ray, could be expressed as:

(Equation 4) $$\theta_{3}=\text{π}-\arcsin\left(n_{2}/n_{1}\right)-2\alpha$$

When *α* is in the range of *β*<*α*<π/2, it could be found that the condition |*θ*_3_|<*θ*_c1_ is satisfied, which means that the upper edge ray could always pass through the interface between the fiber core and cladding.

Similarly, the incident angle of the interface between the fiber core and the air, which corresponds to the bottom edge ray, could be expressed as:

(Equation 5) $$\varphi_{1}=\alpha -\arcsin\left(n_{2}/n_{1}\right)$$

When *α* is in the range of *γ*≤*α*<π/2, in which *γ* is set to be π/4+*θ*_c1_/2, according to Equations 1, 2, and 5, *φ*_1_ would be in the range of *β*/2≤*φ*_1_<*β*, which means that part of the rays near the bottom edge ray would be reflected upwards instead of downwards by the end face, and the percentage of the power, reflected upwards, in the whole reflected light power, varies from 0 to less than 50% as *α* varies from *γ* to being close to π/2 rad.^7^ It would decrease the transmission efficiency of the side-firing power. To avoid this problem, the range of *α* should be 0<*α*<*γ*.

The incident angle of the interface between the fiber core and cladding, which corresponds to the bottom edge ray, could be expressed as:

(Equation 6) $$\varphi_{3}=\arcsin\left(n_{2}/n_{1}\right)-2\alpha$$

To make the bottom edge ray be able to pass through the interface between the fiber core and cladding, *φ*_3_ should satisfy the condition |*φ*_3_|<*θ*_c1_, which means that *α* should be in the range of 0<*α*<*θ*_c1_. Since *θ*_c1_ is in the range of π/4<*θ*_c1_<π/2 mentioned above, *θ*_c1_ is less than *γ*. Therefore, 0<*α*<*θ*_c1_ is included in the range of 0<*α*<*γ*, which means that in this case, there is no ray reflected upwards by the end face. Combining with the range of *β*<*α*<π/2 mentioned above, *α* should be in the range of *β*<*α*<*θ*_c1_, which all the following discussion will be limited to. Here, the two crucial angles *β* and *θ*_c1_ are set as *α*_0_ and *α*_9_, respectively.

The incident angle of the interface between the fiber cladding and the silica cap, which corresponds to the upper edge ray and is equal to the refraction angle *θ*_4_, could be expressed as:

(Equation 7) $$\theta_{5}=\arcsin\left\{n_{1}\;\sin\left[\arcsin\left(n_{2}/n_{1}\right)+2\alpha \right]/n_{2}\right\}$$

The incident angle of the interface between the silica cap and the air, which corresponds to the upper edge ray and is equal to the refraction angle *θ*_6_, could be expressed as:

(Equation 8) $$\theta_{7}=\arcsin\left\{n_{1}\;\sin\left[\arcsin\left(n_{2}/n_{1}\right)+2\alpha \right]/n_{3}\right\}$$

where *n*_3_ is the refractive index of the silica cap. The crucial angle of the interface between the silica cap and the air could be expressed as:^15^

(Equation 9) $$\theta_{\text{c}2}=\arcsin\left(1/n_{3}\right)$$

Since *n*_3_ decreases as the wavelength of the laser increases, *θ*_c2_ increases as the wavelength increases.^15^^,^^25^^,^^26^ To ensure the upper edge ray being able to pass through this interface, the condition |*θ*_7_|<*θ*_c2_ should be satisfied; therefore, according to Equations 8 and 9, the range of *α* should satisfy π/2-arcsin(1/*n*_1_)/2-arcsin(*n*_2_/*n*_1_)/2<*α*<π/2+arcsin(1/*n*_1_)/2-arcsin(*n*_2_/*n*_1_)/2. Here, the two crucial angles π/2-arcsin(1/*n*_1_)/2-arcsin(*n*_2_/*n*_1_)/2 and π/2+arcsin(1/*n*_1_)/2-arcsin(*n*_2_/*n*_1_)/2 are set as *α*_2_ and *α*_8_, respectively. The refraction angle of the interface between the silica cap and the air, which corresponds to the upper edge ray, could be expressed as:

(Equation 10) $$\theta_{8}=\arcsin\left\{n_{1}\;\sin\left[\arcsin\left(n_{2}/n_{1}\right)+2\alpha \right]\right\}$$

According to Equation 10, when the emitted ray, originating from the upper edge ray, is toward the right side of the normal line, which corresponds to *θ*_8_ >0, the condition *α*_2_<*α*<π/2-arcsin(*n*_2_/*n*_1_)/2 should be satisfied. On the other side, when that emitted ray is toward the left side of the normal line, which corresponds to *θ*_8_<0, the condition π/2-arcsin(*n*_2_/*n*_1_)/2<*α*<*α*_8_ should be satisfied. In particular, when that emitted ray is perpendicular to the interface between the silica cap and the air, which corresponds to *θ*_8_=0, the condition *α*=π/2-arcsin(*n*_2_/*n*_1_)/2 should be satisfied. Here, the crucial angle π/2-arcsin(*n*_2_/*n*_1_)/2 is set as *α*_5_.

The incident angle of the interface between the fiber cladding and the silica cap, which corresponds to the bottom edge ray and is equal to the refraction angle *φ*_4_, could be expressed as:

(Equation 11) $$\varphi_{5}=\arcsin\left\{n_{1}\;\sin\left[\arcsin\left(n_{2}/n_{1}\right)-2\alpha \right]/n_{2}\right\}$$

The incident angle of the interface between the silica cap and the air, which corresponds to the bottom edge ray and is equal to the refraction angle *φ*_6_, could be expressed as:

(Equation 12) $$\varphi_{7}=\arcsin\left\{n_{1}\;\sin\left[\arcsin\left(n_{2}/n_{1}\right)-2\alpha \right]/n_{3}\right\}$$

To ensure the bottom edge ray being able to pass through this interface, the condition |*φ*_7_|<*θ*_c2_ should be satisfied. Therefore, according to Equations 9 and 12, the range of *α* should satisfy arcsin(-1/*n*_1_)/2+arcsin(*n*_2_/*n*_1_)/2<*α*<arcsin(1/*n*_1_)/2+arcsin(*n*_2_/*n*_1_)/2. Here, the two crucial angles arcsin(-1/*n*_1_)/2+arcsin(*n*_2_/*n*_1_)/2 and arcsin(1/*n*_1_)/2+arcsin(*n*_2_/*n*_1_)/2 are set as *α*_1_ and *α*_6_, respectively. The refraction angle of the interface between the silica cap and the air, which corresponds to the bottom edge ray, could be expressed as:

(Equation 13) $$\varphi_{8}=\arcsin\left\{n_{1}\;\sin\left[\arcsin\left(n_{2}/n_{1}\right)-2\alpha \right]\right\}$$

According to Equation 13, when the emitted ray, originating from the bottom edge ray, is toward the left side of the normal line, which corresponds to *φ*_8_<0, the condition arcsin(*n*_2_/*n*_1_)/2<*α*<*α*_6_ should be satisfied. On the other side, when that emitted ray is toward the right side of the normal line, which corresponds to *φ*_8_>0, the condition *α*_1_<*α*<arcsin(*n*_2_/*n*_1_)/2 should be satisfied. Particularly, when that emitted ray is perpendicular to the interface between the silica cap and the air, which corresponds to *φ*_8_=0, the condition *α*=arcsin(*n*_2_/*n*_1_)/2 should be satisfied. Here, the crucial angle arcsin(*n*_2_/*n*_1_)/2 is set as *α*_4_.

#### Emitted beam’s divergence angle

According to Equations 10 and 13, the divergence angle of the emitted beam in the fiber axis direction,^12^ could be expressed as:

(Equation 14) $$\Omega =\theta_{8}-\varphi_{8}=\arcsin\left\{n_{1}\;\sin\left[\arcsin\left(n_{2}/n_{1}\right)+2\alpha \right]\right\}-\arcsin\left\{n_{1}\;\sin\left[\arcsin\left(n_{2}/n_{1}\right)-2\alpha \right]\right\}$$

And *Ω* reflects the divergent degree of the emitted beam. Then, the derivative of *Ω* with respect to *α* could be expressed as:

(Equation 15) $$\Omega^{\prime }=\frac{2n_{1}\;\cos\;\left[\arcsin\left(\frac{n_{2}}{n_{1}}\right)+2\alpha \right]}{\sqrt{1-{n_{1}}^{2}\;\sin^{2}\left[\arcsin\left(\frac{n_{2}}{n_{1}}\right)+2\alpha \right]}}+\frac{2n_{1}\;\cos\;\left[\arcsin\left(\frac{n_{2}}{n_{1}}\right)-2\alpha \right]}{\sqrt{1-{n_{1}}^{2}\;\sin^{2}\left[\arcsin\left(\frac{n_{2}}{n_{1}}\right)-2\alpha \right]}}$$

When *α* is π/4 rad, *Ω*′ equals to 0, and it corresponds to the extreme point of *Ω*, which could be expressed as:

(Equation 16) $$\Omega_{0}=2\;\arcsin\left\{n_{1}\;\cos\left[\arcsin\left(n_{2}/n_{1}\right)\right]\right\}$$

On the other side, the quantity of the rays, which are of total internal reflection on the beveled end face, is one of the key factors that affect the final transmission efficiency, and it depends on the crucial angle of the interface between the fiber core and the air, which is at the beveled end face.^7^^,^^8^ This crucial angle could be expressed as:^7^^,^^8^

(Equation 17) $$\theta_{\text{c}3}=\arcsin\left(1/n_{1}\right)$$

Since *n*_1_ decreases as the wavelength of the laser increases, *θ*_c3_ increases as the wavelength increases.^15^^,^^25^^,^^26^ When all the rays in the cone are totally internally reflected, the condition *φ*_1_≤-*θ*_c3_ should be satisfied, therefore, the range of *α* should satisfy *α*≤arcsin(*n*_2_/*n*_1_)-arcsin(1/*n*_1_); on the contrary, when no total internal reflection happens, the condition *θ*_1_>-*θ*_c3_ is satisfied, and in this case, the range of *α* satisfies *α*>π-arcsin(*n*_2_/*n*_1_)-arcsin(1/*n*_1_).^8^ Here, the two crucial angles arcsin(*n*_2_/*n*_1_)-arcsin(1/*n*_1_) and π-arcsin(*n*_2_/*n*_1_)-arcsin(1/*n*_1_) are set as *α*_3_ and *α*_7_, respectively.

When *α* is larger than *α*_3_, the total internal reflection is not achieved for all the rays in the cone, and part of the light would pass through the beveled end face; in this case, the beam may propagate both from the front and the side of the fiber, which would decrease the transmission efficiency.^5^^,^^8^^,^^10^^,^^22^^,^^23^

#### Emitted beam’s coverage

The variation of the ray’s optical path with *α* may cause the fluctuation of the emitted beam’s coverage, which directly affects the ablation range.^1^^,^^4^ Here, in the direction of the fiber axis, how the coverage varies with *α* is studied on the outer surface of the metal cap, which may be very close to the target tissue.^1^^,^^4^^,^^13^^,^^14^^,^^16^ In the direction of the fiber axis, the deviation for A from O could be expressed as:

(Equation 18) $$L_{1}=m+\left(d_{1}/2-m\text{tg}\alpha \right)\mathrm{tg}\theta_{3}+\left(d_{2}-d_{1}\right)\mathrm{tg}\theta_{5}/2+\left(d_{3}-d_{2}\right)\mathrm{tg}\theta_{7}/2+\left(d_{4}-d_{3}\right)\mathrm{tg}\theta_{8}/2$$

where *m* is the deviation of the upper edge ray’s incident point on the beveled end face from O, which is in the direction of the fiber axis; *d*_1_ and *d*_2_ are the diameters of the fiber core and cladding, respectively; *d*_3_ and *d*_4_ are the outer diameters of the silica and metal caps, respectively. Here, *m* is positive and negative for the upper edge ray’s incident point being on the right and left of O, respectively; *L*_1_ is positive and negative for A being on the right and left of O, respectively. Particularly, when the upper edge ray’s incident point is at the bottom edge of the beveled end face within the fiber core, *m* could be expressed as:

(Equation 19) $$m=d_{1}/\left(2\mathrm{tg}\alpha \right)$$

Then, the maximum of *L*_1_ could be expressed as:

(Equation 20) $$L_{1\text{max}}=d_{1}/\left(2\mathrm{tg}\alpha \right)+\left(d_{2}-d_{1}\right)\mathrm{tg}\left(\arcsin\left\{n_{1}\;\sin\left[\arcsin\left(n_{2}/n_{1}\right)+2\alpha \right]/n_{2}\right\}\right)/2+\left(d_{3}-d_{2}\right)\mathrm{tg}\left(\arcsin\left\{n_{1}\;\sin\left[\arcsin\left(n_{2}/n_{1}\right)+2\alpha \right]/n_{3}\right\}\right)/2+\left(d_{4}-d_{3}\right)\mathrm{tg}\left(\arcsin\left\{n_{1}\;\sin\left[\arcsin\left(n_{2}/n_{1}\right)+2\alpha \right]\right\}\right)/2$$

The deviation for B from O, which is in the direction of the fiber axis, could be expressed as:

(Equation 21) $$L_{2}=n-\left(d_{1}/2+n\text{tg}\alpha \right)\mathrm{tg}\varphi_{3}-\left(d_{2}-d_{1}\right)\mathrm{tg}\varphi_{5}/2-\left(d_{3}-d_{2}\right)\mathrm{tg}\varphi_{7}/2-\left(d_{4}-d_{3}\right)\mathrm{tg}\varphi_{8}/2$$

where *n* is the deviation of the bottom edge ray’s incident point on the beveled end face from O, which is in the direction of the fiber axis. Here, *n* is positive and negative for the bottom edge ray’s incident point being on the left and right of O, respectively; *L*_2_ is positive and negative for B being on the left and right of O, respectively. In particular, when the bottom edge ray’s incident point is at the upper edge of the end face within the fiber core, *n* could be expressed as:

(Equation 22) $$n=d_{1}/\left(2\mathrm{tg}\alpha \right)$$

Then, the maximum of *L*_2_ could be expressed as:

(Equation 23) $$L_{2\max}=d_{1}/\left(2\mathrm{tg}\alpha \right)-d_{1}\mathrm{tg}\left[\arcsin\left(n_{2}/n_{1}\right)-2\alpha \right]-\left(d_{2}-d_{1}\right)\mathrm{tg}\left(\arcsin\left\{n_{1}\;\sin\left[\arcsin\left(n_{2}/n_{1}\right)-2\alpha \right]/n_{2}\right\}\right)/2-\left(d_{3}-d_{2}\right)\mathrm{tg}\left(\arcsin\left\{n_{1}\;\sin\left[\arcsin\left(n_{2}/n_{1}\right)-2\alpha \right]/n_{3}\right\}\right)/2-\left(d_{4}-d_{3}\right)\mathrm{tg}\left(\arcsin\left\{n_{1}\;\sin\left[\arcsin\left(n_{2}/n_{1}\right)-2\alpha \right]\right\}\right)/2$$

According to Equations 20 and 23, the distance *L* shown in Figure 2 could be expressed as:

(Equation 24) $$L=L_{1\max}+L_{2\max}$$

#### Crucial value of *n*_2_

From Figure 3, it could be found that the relationship among these crucial angles would be *α*_0_<*α*_1_<*α*_2_<*α*_3_<*α*_4_<*α*_5_<*α*_6_≤*α*_7_<*α*_8_<*α*_9_ and *α*_0_<*α*_1_<*α*_2_<*α*_3_<*α*_4_<*α*_5_<*α*_7_<*α*_6_<*α*_8_<*α*_9_ corresponding to *n*_2_ being not larger and larger than a certain value *n*_20_ respectively, and by solving *α*_6_=*α*_7_, *n*_20_ could be expressed as:

(Equation 25) $$n_{20}=n_{1}\;\sin\left[2\text{π}/3-\arcsin\left(1/n_{1}\right)\right]$$

As mentioned above, the suitable wavelength for the laser would be from about 500 nm to 2300 nm; therefore, the value of *n*_1_ would be in the range from about 1.464 to 1.433,^26^ and *n*_20_ decreases as the wavelength increases. Calculated by Equation 25, *n*_20_ would not be larger than 1.426 but not be less than 1.389, and particularly, *n*_20_ is about 1.418 with *n*_1_=1.457.

#### Increase rate for *Ω* and *L*

When *α* deviates from π/4 rad for *ε*, according to Equation 14, *Ω* could be expressed as:

(Equation 26) $$\Omega =\arcsin\left\{n_{1}\;\cos\left[\arcsin\left(n_{2}/n_{1}\right)+2\varepsilon \right]\right\}+\arcsin\left\{n_{1}\;\cos\left[2\varepsilon -\arcsin\left(n_{2}/n_{1}\right)\right]\right\}$$

Therefore, the increase rate for *Ω* could be expressed as:

(Equation 27) $$\eta =\left(\Omega -\Omega_{0}\right)/\Omega_{0}=\left(\arcsin\left\{n_{1}\;\cos\left[\arcsin\left(n_{2}/n_{1}\right)+2\varepsilon \right]\right\}+\arcsin\left\{n_{1}\;\cos\left[2\varepsilon -\arcsin\left(n_{2}/n_{1}\right)\right]\right\}-2\;\arcsin\left\{n_{1}\;\cos\left[\arcsin\left(n_{2}/n_{1}\right)\right]\right\}\right)/\left(2\;\arcsin\left\{n_{1}\;\cos\left[\arcsin\left(n_{2}/n_{1}\right)\right]\right\}\right)$$

When *α* is at a certain value *α*_m_, which is close to π/4 rad, *L* reaches its minimum, and this minimum could be expressed as:

(Equation 28) $$L_{0}=L\left(\alpha =\alpha_{\text{m}}\right)$$

When *α* deviates from *α*_m_ for *ε*, according to Equation 24, *L* could be expressed as:

(Equation 29) $$L=L\left(\alpha =\alpha_{m}+\varepsilon \right)$$

Then, the increase rate for *L* could be expressed as:

(Equation 30) $$\zeta =\left(L-L_{0}\right)/\;L_{0}$$

#### Distance between emitted edge ray and O

As mentioned above, the spot of the emitted beam could be assumed to be a symmetric elliptical shape, and the length of this elliptical shape, in the direction of the fiber axis, varies with *α* and the distance from the surface of the metal cap.^5^^,^^6^^,^^7^^,^^8^^,^^9^^,^^12^^,^^13^^,^^14^ In the direction of the fiber axis, the distance between the emitted ray and O, originating from the upper edge ray, could be expressed as:

(Equation 31) $$r_{1}=L_{1\max}+Q\mathrm{tg}\theta_{8}$$

where *Q* is the distance from the metal cap’s surface. And the distance between the emitted ray and O, originating from the bottom edge ray, could be expressed as:

(Equation 32) $$r_{2}=-L_{2\max}+Q\mathrm{tg}\varphi_{8}$$

The sign of *r*_1_ or *r*_2_ is positive and negative corresponding to the emitted ray being on the right and left of O respectively.

#### Emission opening’s required size

To avoid the emitted rays being blocked, the edge dimension of the metal cap’s emission opening should be designed according to the emitted beam’s optical field distribution range. Since the metal cap’s tube wall has a certain thickness, according to the geometrical relationship, the expression for the designed dimension of the emission opening’s edge would be different when the emitted edge rays are toward the different sides of the normal line, which is decided by the range of *α*. When that factor is considered, the crucial distance *D*_1_ shown in Figure 2 could be expressed as:

(Equation 33) $$D_{1}=\left\{ \begin{array}{c} L_{1\max}\;\left(\alpha_{2}<\alpha \leq \alpha_{5}\right)\\ L_{1\max}-\left(d_{4}-d_{3}\right)\text{tg}\theta_{8}/2\;\left(\alpha_{5}<\alpha <\alpha_{6}\right) \end{array}\right.$$

And the crucial distance *D*_2_ shown in Figure 2 could be expressed as:

(Equation 34) $$D_{2}=\left\{ \begin{array}{c} L_{2\max}+\left(d_{4}-d_{3}\right)\text{tg}\varphi_{8}/2\;\left(\alpha_{2}<\alpha \leq \alpha_{4}\right)\\ L_{2\max}\;\left(\alpha_{4}<\alpha <\alpha_{6}\right) \end{array}\right.$$

Considering the machining tolerance, to ensure no emitted ray being blocked, according to the monotonicity of *D*_1_ and *D*_2_ shown in Figure 11, the actual value for *D*_1_ should be:

(Equation 35) $${D_{1}}^{'}=\left\{ \begin{array}{c} L_{1\max}\;\left(\alpha =\alpha -\upsilon \right)\;\left(\alpha_{2}+\upsilon <\alpha \leq \alpha_{5}+\upsilon \right)\\ L_{1\max}\left(\alpha =\alpha -\upsilon \right)-\left(d_{4}-d_{3}\right)\text{tg}\left[\theta_{8}\left(\alpha =\alpha -\upsilon \right)\right]/2\;\left(\alpha_{5}+\upsilon <\alpha <\alpha_{6}-\sigma \right) \end{array}\right.$$

And the actual value for *D*_2_ should be:

(Equation 36) $${D_{2}}^{'}=\left\{ \begin{array}{c} L_{2\max}\left(\alpha =\alpha +\sigma \right)+\left(d_{4}-d_{3}\right)\text{tg}\left[\varphi_{8}\left(\alpha =\alpha +\sigma \right)\right]/2\;\left(\alpha_{2}+\upsilon <\alpha \leq \alpha_{4}-\sigma \right)\\ L_{2\max}\;\left(\alpha =\alpha +\sigma \right)\;\left(\alpha_{4}-\sigma <\alpha <\alpha_{6}-\sigma \right) \end{array}\right.$$

Here, to ensure the actual value of *α* being less than *α*_6_ and larger than *α*_2_, it is noted that the whole range of the designed *α* is further limited to being *α*_2_+*υ*<*α*<*α*_6_-*σ*. Then, the actual crucial distance between the right and left edges of the emission opening could be expressed as:

(Equation 37) $$D^{'}=\left\{ \begin{array}{c} L_{1\max}\;\left(\alpha =\alpha -\upsilon \right)+L_{2\max}\left(\alpha =\alpha +\sigma \right)+\left(d_{4}-d_{3}\right)\text{tg}\left[\varphi_{8}\left(\alpha =\alpha +\sigma \right)\right]/2\;\left(\alpha_{2}+\upsilon <\alpha \leq \alpha_{4}-\sigma \right)\\ L_{1\max}\;\left(\alpha =\alpha -\upsilon \right)+L_{2\max}\;\left(\alpha =\alpha +\sigma \right)\;\left(\alpha_{4}-\sigma <\alpha \leq \alpha_{5}+\upsilon \right)\\ L_{1\max}\left(\alpha =\alpha -\upsilon \right)-\left(d_{4}-d_{3}\right)\text{tg}\left[\theta_{8}\left(\alpha =\alpha -\upsilon \right)\right]/2+L_{2\max}\;\left(\alpha =\alpha +\sigma \right)\;\left(\alpha_{5}+\upsilon <\alpha <\alpha_{6}-\sigma \right) \end{array}\right.$$

And the practical selected distance should be not less than *D*’ while being as close to *D*’ as possible. In practice, besides the direction considered in this paper, it is better that the opening could precisely match the allowed amount of light in all directions. The reasons are as follows. Firstly, it may ensure no allowed emitted ray being blocked, which originates from the cone; secondly, it may better shield any laser energy reflected back from the target tissue; thirdly, it may shield any internal scattered light from escaping, which originates from the rays reflected by such as the inner surface of the metal cap.^11^^,^^13^^,^^14^
